# Addition of Carrot Pomace to Enhance the Physical, Sensory, and Functional Properties of Beef Patties

**DOI:** 10.3390/foods13233910

**Published:** 2024-12-03

**Authors:** Jordan Richards, Amy Lammert, Jack Madden, Anna Cahn, Iksoon Kang, Samir Amin

**Affiliations:** 1 Food Science and Nutrition Department, California Polytechnic State University, San Luis Obispo, CA 93407, USA; jrichards@batoryfoods.com (J.R.); alammert@calpoly.edu (A.L.); jmmadden@calpoly.edu (J.M.); acahn@calpoly.edu (A.C.); 2 Animal Science Department, California Polytechnic State University, San Luis Obispo, CA 93407, USA; ikang01@calpoly.edu

**Keywords:** food waste, carrot pomace, beef patty, dietary fiber, functional properties, sensory evaluation, valorization

## Abstract

The global challenge of food waste necessitates innovative solutions, such as incorporating carrot pomace, a nutrient-rich by-product of carrot juice production, into beef patties to enhance their nutritional and functional properties. This study evaluated beef patties with carrot pomace added at 0%, 1.0%, 3.0%, and 4.2%, analyzing the proximate composition, pH, color, cooking yield, water-holding capacity (WHC), texture, and sensory attributes. Adding 3.0% and 4.2% pomace significantly reduced the moisture content by 5.5% and 3.3%, respectively, and decreased redness by 40% in the 4.2% patties. The cooking yield increased by 13.9% and 22.8%, and WHC improved by 8.5% and 15.7%, respectively, with these additions. The textural properties showed substantial reductions in hardness, cohesiveness, gumminess, and chewiness, particularly at 4.2%. The sensory evaluation indicated no significant differences in appearance, aroma, taste, or overall liking for patties with up to 3% pomace. In comparison, patties with 4.2% pomace received lower scores for overall liking and firmness. These findings suggest that incorporating up to 3% carrot pomace in beef patties enhances their functional properties and dietary fiber content without compromising the sensory quality. This offers a sustainable and practical approach to food waste valorization.

## 1. Introduction

Approximately one-third of all food produced globally is lost or wasted somewhere along the food supply chain. Food loss and waste occur throughout the entire food processing cycle, from in-field harvest to processing and packaging facilities, and even at grocery retail stores. This represents a waste of the water, land, energy, and natural resources used to produce the foods, causing USD 940 billion in economic losses and over 4.4 gigatons of greenhouse gas emissions (CO_2_ equivalent) annually [1]. The United States Environmental Protection Agency (EPA) estimates that the annual food loss and waste is equivalent to 170 million metric tons of CO_2_ equivalent emissions within the U.S. [2]. Reducing food waste within the U.S. presents opportunities to address climate change, conserve natural resources, increase food security, and enhance economic efficiency. According to the United States Department of Agriculture (USDA), 31% of food is wasted at a cost of USD 218 billion or 1.3% of the Gross Domestic Product (GDP) [3].

Over the past 35 years, the U.S. carrot industry has been transformed by the introduction of fresh-cut technology, leading to the production of more value-added carrot products, such as pre-cut carrots, baby carrots, and carrot juice. However, this shift has also increased the amount of waste generated from carrot processing. In carrot juice production, the pulp by-product known as carrot pomace constitutes to 50% of the raw material [4]. Carrot pomace is rich in bioactive compounds, particularly carotenoids, such as β-carotene [5,6]. It is also rich in both insoluble and soluble fibers, with the ideal levels of pectic polysaccharides, hemicellulose, and cellulose [4,6].

The consumption of dietary fiber is known to promote bowel movements and the feeling of satiety [7]. As an essential nutrient, dietary fiber also helps reduce the risks of chronic diseases, including heart disease and diabetes; it can also improve cholesterol levels and reduce blood pressure [8]. These substances play a pivotal role in food manufacturing as insoluble (cellulose, hemicellulose, and lignin) and soluble (pectin and other gums) fibers can affect the water-holding capacity, gel-forming ability, and fat-binding capacity of dietary fiber. Carrot pomace contains approximately 55% total dietary fiber on a dry weight basis [9]. Previously, several attempts have been made to utilize carrot pomace in food products due to its functional, nutritional, and health-promoting benefits [4,10,11]. These beneficial factors make carrot pomace a promising ingredient for in value-added products, especially in the meat industry.

Humans have evolved and adapted to consuming large quantities of red meat and poultry [12]. In 2020, the total production of meat in the U.S. was 48.7 million tons, which increased from 22.1 million tons in 1971 to 48.7 million tons in 2020, with an average annual growth rate of 1.65% [13]. The total per capita meat consumption has steadily increased, with an overall average increase of 2.5 lbs/year, resulting in 226.9 lbs consumed on average per person in 2021 [14]. Red meat is an important dietary source of protein and essential nutrients, including iron, zinc, and vitamin B12. Several factors, such as annual income, livestock production capacity, and the socioeconomic status of consumers, could explain the higher consumption of meat in Western countries [15].

Meat and meat products have both positive and negative effects on human health. They are important sources of protein, essential amino acids and fats, vitamins A and B, and minerals [16]. However, fresh and processed meat products have been viewed negatively due to their high levels of saturated fats, cholesterol, sodium, and undesirable ingredients, including sodium nitrite [17]. There are ongoing efforts to address these concerns by using leaner meat, minimizing processing, and reducing or replacing sodium and nitrites [18]. Meat and meat products lack dietary fiber, which could be fortified by a simple addition of high-fiber ingredients, such as carrot pomace. The long-term and high consumption of meat and meat products, especially processed meat products, has been linked to an increased risk of cardiovascular disease, obesity, type 2 diabetes, and certain types of cancers [19]. Incorporating dietary fiber with meat products could help mitigate those kinds of risk factors [8].

Numerous studies have evaluated the effects of incorporating dietary fiber in various meat products carrot fiber in fermented sausage and pork sausage [20,21]; oat flour in cooked kofta [22]; oat bran in chicken patties [23]; wheat fiber in sausages [24]; wheat bran in meatballs, beef patties, and chicken patties [23,25,26]; pea fiber in beef patties and chicken nuggets [27,28]; grape fiber in chicken breast patties [29]; and apple pulp in chicken nuggets [30]. The incorporation of dietary fiber also increased the cooking yield and fat reduction due to its water-holding and fat-binding properties [25,26,27,31].

Utilizing carrot pomace presents an opportunity to address food waste issues while providing a functional ingredient to the food industry. The objective of this study is to evaluate the potential of incorporating freeze-dried carrot pomace, a nutrient-rich by-product of carrot juice production, into beef patties to enhance their nutritional and functional properties while addressing food waste. Specifically, the research aims to analyze the impact of varying levels of carrot pomace (0%, 1.0%, 3.0%, and 4.2%) on the patties’ proximate composition, physical analysis, and sensory attributes to determine the optimal inclusion level for maintaining consumer acceptability and improving product functionality.

## 2. Materials and Methods

Carrot pomace was obtained from Grimmway Family Farms (Arvin, CA, USA). The pomace was placed in 22 kg sealed, food-grade pails, and stored in a dark freezer at −20 °C until further processing. Freezing pomace prior to processing minimized the chances of microbial growth and the degradation of carotenoids.

### 2.1. Mechanical Drying Treatment of Carrot Pomace

Carrot pomace was freeze-dried (FD) using a freeze dryer (Harvest Right Freeze Dryer) at −20 °C for 24 h. The dried carrot pomace was ground using a commercial spice grinder (VEVOR 2500 g Electric Grain Mill Grinder, Sacramento, CA, USA) to pass through a 20-mesh sieve (0.85 mm). Dried and ground carrot pomace was stored in plastic bags wrapped in aluminum foil at −22 °C.

### 2.2. Development of Beef Patties with Carrot Pomace

Beef round and beef fat were procured from the Cal Poly Meat Processing Center (MPC). Ground beef was prepared by grinding a mixture of 80% beef round and 20% beef fat using a 3/8″ (9.5 mm) diameter plate (BigBite^®^ Meat Grinder, LEM, West Chester, OH, USA). This mixture was then combined with 0%, 1%, 3%, and 4.2% freeze-dried carrot pomace and further ground through a 3/16″ (4.76 mm) diameter plate. Additional ingredients were then manually mixed according to Table 1. The resulting mixture was weighed and formed into 60 g patties (7 cm dia.) using a patty press (513 Mini Burger Press, Norpro, Everett, WA, USA). The patties were pre-frozen on trays overnight at −20 °C, vacuum-sealed, and stored at −20 °C.

#### 2.2.1. Proximate Analysis

Raw beef patty samples containing 0%, 1%, 3%, and 4.2% carrot pomace were analyzed for proximate composition on a dry basis [32]. Samples were pre-weighted and placed in an HFS vacuum oven (Model DZF-6050, HFS Inc., Azusa, CA, USA) and dried under vacuum at 70 °C for 16 h. The set temperature was selected to prevent the oxidation of fatty acids due to the high-fat content of the beef mixture, which could otherwise lead to weight gain in the samples, affecting accuracy. Samples were removed and weighed, and the loss from drying was used to determine the moisture content. The dried samples were used to determine the protein, fat, moisture, and ash contents. Samples were analyzed in triplicate.

The protein content was determined using the Kjeldahl method (FOSS Tecator™ Digestor, FOSS Kjeltec™ 8200 Auto Distillation Unit, Eden Prairie, MN, USA) to determine the percentage of nitrogen in the sample (AOAC 981.10). A standard conversion factor of 6.25, typical for food products, was used to calculate the protein content.

The fat content was determined using Soxhlet extraction (FOSS Soxtec™ 2043, Eden Prairie, MN, USA) with petroleum ether (AOAC 991.36). The samples were used to conduct ash analysis by placing them in a muffle furnace (Barnstead Thermolyne 62700, Thermolyne Corporation, Dubuque, IA, USA) at 600 °C for 24 h. Total carbohydrates were calculated by difference using the following equation [33]:

Total Carbohydrate (%) = 100 − (Protein % + Fat % + Ash %)

#### 2.2.2. pH of Uncooked Beef Patty

Raw beef patty (control and carrot pomace samples) (5.00 g) were blended with 15.00 mL distilled water for 30 s with a Vitamix Professional Series 500 blender (Cleveland, OH, USA). The pH was measured in triplicate with an Orion Star A211 Benchtop pH meter (Thermo Scientific, Waltham, MA, USA) calibrated using pH 3 and 7 standards.

#### 2.2.3. Water-Holding Capacity of Uncooked Beef Patty

The water-holding capacity of the uncooked beef patties was determined according to the method by Hughes [34] with modifications. A 30.0 g sample of each beef patty having 0%, 1%, 3%, or 4.2% carrot pomace was weighed and mixed with 10 g of water using a kitchen aid mixer, and then placed into 50 mL centrifuge tubes. The tubes were heated in a 90 °C water bath for 10 min until the internal temperature of the product reached 165 °F. After cooling to room temperature, the samples were centrifuged (Eppendorf 5810 R Centrifuge, Hauppauge, NY, USA) at 1400 rpm (270× *g*) for 15 min and then chilled in the refrigerator for 24 h. On the next day water, the fat and meat were separated and weighed. Samples were analyzed in triplicate. The water-holding capacity (WHC) of the beef patties was calculated using the equation below:

$$\mathrm{Water Holding Capacity}=1-\frac{\left(\mathrm{weight}\;1-\mathrm{weight}\;2\right)}{\mathrm{Moisture}\%}\times 100$$

Weight 1 = weight of beef + water pre centrifugation

Weight 2 = weight of sample after centrifugation

#### 2.2.4. Color of Uncooked Beef Patty

The color of the raw beef patty (control and carrot pomace samples) was analyzed using a WR10—8 Series Calorimeter (FRU Industries, Longhua New Area, Shenzhen, China). L* values represent the lightness in the range from 0 (black) to 100 (white); a* values indicate the shift from green (−) to red (+), and b* values measure the change from blue (−) to yellow (+). A total of 10 raw beef patties were analyzed and color measurements were taken in three different areas (left, middle, and right) of each patty. The total color difference (ΔE) was calculated using the following equation, with the control patty (0% carrot pomace) serving as a reference.

ΔE = [(ΔL*)^2^ + (Δa*)^2^ + (Δb*)^2^]^1/2^

ΔE = Total color difference

ΔL = L*sample − L*ref

Δa* = a*sample − a*ref

Δb* = b*sample − b*ref

ΔE can be interpreted as the following [35]:

0 < ΔE < 1: the difference is not noticeable;

1 < ΔE < 2: experienced observers may notice the difference;

2 < ΔE < 3.5: inexperienced observers may notice a difference;

3.5 < ΔE < 5: a clear color difference is noticed;

5 < ΔE: observers notice two different colors.

#### 2.2.5. Cooking Yield of Beef Patty

Frozen patties were cooked using an Avantco P70S Commercial Grill (Clark Associates Inc., Lancaster, PA, USA) preheated to 163 °C, without adding fat, until they reached an internal temperature of 71 °C. Patties were allowed to cool to 20 °C prior to testing. Samples were analyzed in triplicate. Patties were weighed before and after cooking to determine the cooking yield using the equation below.

$$\mathrm{Cooking Yield}(\%)=\frac{\mathrm{weight of cooked patty}\;(g)}{\mathrm{weight of uncooked patty}\;(g)}\times 100$$

#### 2.2.6. Texture Analysis of Cooked Beef Patty

Frozen patties were cooked using an Avantco P70S Commercial Grill (Clark Associates Inc., Lancaster, PA, USA) preheated to 163 °C, without adding fat, until they reached an internal temperature of 71 °C. Patties were allowed to cool to 20 °C prior to testing. The texture of cooked beef patties with 0%, 1%, 3%, and 4.2% carrot pomace was analyzed using a Brookfield CT3 Texture Analyzer (AMETEK Brookfield, Middleboro, MA, USA). The parameters were set to a trigger load of 7.0 g and speed at 1.70 mm/s for two compressions, using a TA10 cylinder probe (D: 12.7 mm, L: 35 mm). A total of 10 cooked beef patties were analyzed. The following texture profile analysis parameters were determined: hardness-1 (force required for the first compression), hardness-2 (peak force for the second compression), adhesiveness, cohesiveness, springiness (elasticity), gumminess, and chewiness. Hardness is described as the maximum force at the first compression, and adhesiveness is the amount of work it takes to pull the probe out of the product. Cohesiveness describes how well the food product retains its form between the first and second compressions. It is calculated by comparing the area under the second compression curve to the area under the first compression curve, providing a relative, dimensionless measure of the sample’s ability to withstand a second deformation. Springiness is the distance the sample recovered in height after the first compression. Chewiness is the product of hardness-1, springiness, and cohesiveness, whereas gumminess is the product of hardness-1 and cohesiveness [36].

#### 2.2.7. Statistical Analysis

A one-way analysis of variance (ANOVA) was used to determine significant differences between beef patty formulations using the JMP Pro 17 statistics software (Cary, NC, USA). Tukey’s post hoc analysis was performed to identify significant differences between treatments at *p* ≤ 0.05.

### 2.3. Sensory Test of Cooked Beef Patty with Added Carrot Pomace

An IHUT (in-home use test) was conducted to evaluate the beef patties containing carrot pomace at four different levels (0%, 1%, 3%, and 4.2%). The patties were evaluated with and without a bun (Rockenwagner Bakery, Los Angeles, CA, USA). Frozen patties were cooked using an Avantco P70S Commercial Grill (Clark Associates Inc., Lancaster, PA, USA) preheated to 163 °C, without adding fat, until they reached an internal temperature of 71 °C. Cooked patties were chilled to 4 °C under refrigeration. Individual cooled patties were vacuum packed in 7 mil nylon and low-density polyethylene bags and heat-sealed. Packed patties were stored under refrigeration at 4 °C until distribution to participants. All online questionnaires for recruitment and sensory testing were developed and completed using the RedJade sensory software, version 5.1.0 (Martinez, CA, USA).

#### 2.3.1. Ethics Statement

This research was reviewed and approved by the Cal Poly San Luis Obispo Institutional Review Board (2023-012-CP).

#### 2.3.2. Participants and Recruitment

A recruitment email was sent to untrained consumers through the Cal Poly SLO Sensory consumer database in RedJade. The email contained a website link to answer a screener questionnaire. Consumers were qualified for participation if they met the following requirements: age of 18 years and over, no declared food allergies and/or intolerances, frequent consumption of beef at least once a month, willingness to consume food products containing carrots and beef, and ability to come in and pick up the product during the day and time offered for the sensory test. After screening, 121 participants were qualified for the sensory test and were sent a follow-up email with instructions on how to participate. Sample picking occurred on Friday, 21 April 2023, from 9:00 am to 5:00 pm PST, with participants requested to complete the testing by Sunday, 23 April 2023, at 12:00 am PST. Participants received a USD 10 Amazon gift card after completing the test. By the end, 74 pre-qualified and 12 walk-in participants (*n* = 86) completed the sensory evaluation as instructed. In-home use tests (IHUTs) evaluate product performance in natural settings, offering realistic insights and long-term feedback [37]. However, they face challenges like reduced control, limited observation, and participant compliance issues [37]. The IHUT requires a minimum of 40 and a maximum of 300 respondents to be valid [38,39,40].

#### 2.3.3. Product Pick-Up and Procedure

Participants were instructed to collect samples from the Department of Food Science & Nutrition at California Polytechnic State University, San Luis Obispo, during their designated times. Participants were asked to bring ID and a pen to the location. Upon arrival, participants were checked in and provided an insulated lunch pack containing 4 pre-cooked samples (0%, 1%, 3%, and 4.2%), 4 buns, 1 bottle of water, and crackers. Participants were instructed that only they could complete the questionnaire and the RedJade software would remind them to take a bite of a cracker and a sip of water between samples. Participants were also provided packaging slips containing participant sample codes and instructions on how to access the RedJade website, along with written instructions to reheat the samples by heating the sealed pre-cooked beef patties in boiling water (100 °C) for 5 min (See Appendix A). Reheating the sealed, pre-cooked beef patties in boiling water was chosen to provide participants with a consistent method to safely reheat the patties without excessively drying them out. Participants were also informed of an instructional video (Supplementary Materials Video S1) that was sent out prior to sample pick-up.

#### 2.3.4. Consumer Acceptability Testing

After collecting the samples and following the reheating instructions, participants were requested to use 4-digit-coded samples to answer the questionnaire in Red Jade, which would lead them to the next page until completion. Once the sample code was entered, participants responded to the questions that evaluated the products for the following attributes using a 9-point hedonic scale (1 = Dislike Extremely to 9 = Like Extremely): appearance, aroma, overall liking, taste, texture, flavor, and aftertaste. Participants were also asked to evaluate five attributes (color, juiciness, beef flavor, with a bun juiciness, and with a bun beef flavor), using the 5-point just-about-right (JAR) scale. A penalty analysis was obtained using JAR data. The penalty analysis was conducted to confirm which of the attributes affected the acceptability of the product to a greater or lesser extent.

#### 2.3.5. Relative Intensity Change

Relative intensity change in food sensory testing refers to measuring and evaluating variations in sensory attributes, such as taste, aroma, or texture, over time or between samples. To evaluate how the addition of the bun impacted patty liking, relative changes (%) in liking scores were determined by calculating the difference in liking between patties evaluated alone (Lpattyalone) and patties combined with a bun (Lpattywithbun) and dividing the liking difference by the liking of the patties evaluated alone (Lpattyalone) [41].

$$\mathrm{Relative Intensity Change}(\%)=\frac{\mathrm{Lpattyalone}-\mathrm{Lpattywithbun}}{\mathrm{Lpattyalone}}\times 100$$

## 3. Results and Discussion

### 3.1. Development of a Functionally Enhanced Beef Patty

#### 3.1.1. Proximate Analysis of Raw Beef Patties

The moisture content (MC) % of uncooked beef patties ranged from 60.4% to 63.9%. There were no significant differences in MC between the control and 1% pomace beef patties (*p* > 0.05). However, there was a significant decrease in MC between pomace patties at 3 to 4.2% and control patties (*p* ≤ 0.05). The moisture content decreased as the pomace percentage increased (Table 2). The decrease was due to the replacement of meat with dried carrot pomace, which has much lower moisture compared to meat. Similar results were observed in beef patties formulated with dried pumpkin pulp and seeds, where the moisture content decreased from 60.46 to 57.78% as the pumpkin pulp increased from 0 to 5% [42].

There was no significant difference in the protein and ash contents between beef patty samples (*p* > 0.05). Even though the differences were not significant, ash values increased slightly with the addition of carrot pomace, potentially due to the higher ash content in the carrot pomace compared to meat. Similarly, dried carrot pomace incorporated into chicken sausages at 6% and 9% showed an increase in the ash content compared to the control [4].

The fat content of beef patties was not significantly different between samples; however, it decreased as carrot pomace was added at the levels of 3% and 4.2%. These results indicate that the addition of carrot pomace did not cause significant changes in the proximate composition of beef patties. Similar results were seen in dry fermented sausages, where increased levels of carrot dietary fiber led to a decrease in the moisture and fat contents without significant differences in the protein and ash contents [20]. Freeze-dried carrot pomace contains 71.82% dietary fiber, of which 55.38% is insoluble fiber and 16.49% is soluble fiber [6]. The recommended daily dietary fiber is 25 g to 28 g for a 2000-calorie diet. The addition of carrot pomace increases the dietary fiber content in beef patty to 3%. According to the FDA, a product that contains 2.50 g of fiber per serving can be labeled as a “good source of fiber” [43]. Freeze-dried carrot pomace comprises 71.87% total dietary fiber, which includes 55.38% insoluble and 16.49% soluble dietary fiber [6]. Adding freeze-dried carrot pomace increased the amount of dietary fiber compared to the control. Increasing pomace from 1% to 3% increased fiber by 198%, increasing pomace from 1% to 4.2% increased fiber by 317%, and increasing pomace from 3% to 4.2% increased fiber by 40%. The only raw patty that would be considered a “good source of fiber” are the patties with 4.2%. For cooked patties, the 3% and 4.2% additions provide 2.8 g and 3.65 g of fiber per serving, respectively.

#### 3.1.2. pH of Raw Beef Patties

The addition of carrot pomace into beef patties showed no significant differences in pH compared to the control patties (*p* > 0.05), although the pH value decreased from 5.59 to 5.2 as the percentage of pomace increased from 0% to 4.2% (Table 2). The decrease in pH as the percentage of pomace increased can be attributed to the addition of the pomace. The pH values in meat are an important factor in the water-holding capacity. As the pH values approach the isoelectric point of proteins (5.4), more moisture loss occurs due to less electric charges and protein denaturation [44]. The pH results for the control and 1% patties were within the normal pH range (5.4–5.7) for raw, unstressed bovine animal protein [45]. The pH of the fiber source can influence the pH of the meat product. For example, the pH of chicken sausages increased significantly with the addition of wheat bran, while the addition of dried carrot pomace resulted in a decrease [4]. Similarly, the pH value of raw burgers decreased with the addition of passion fruit albedo [46]. These effects should be considered when formulating patties with low pH ingredients, as they impact the overall pH of the meat product.

#### 3.1.3. Water-Holding Capacity of Beef Patties

The water-holding capacity of control beef patties ranged between 75.08 and 89.16% (Table 2). The incorporation of carrot pomace for 4.2% increased the WHC by 15.67% compared to the control (*p* ≤ 0.05). As the percentage of carrot pomace increased, a positive effect on the WHC of beef patties was observed at the 4.2% level. The high water-holding capacity of carrot pomace is likely responsible for this increase. Beef patties formulated with up to 5% pumpkin pulp also showed a significant increase in WHC [42]. The addition of wheat fiber to beef burgers showed a similar pattern due to the high water-holding capacity of the fiber [47].

#### 3.1.4. Cook Yield of Cooked Beef Patties

The cooking yield of beef patties increased significantly by 9% and 15%, with the addition of carrot pomace by 3% and 4.2%, respectively (Table 2). These results were expected due to the water-holding capacity and fat-binding ability of carrot pomace [6]. The yield of meat cuts and meat products is linked with the amount of fat and water retention [48]. The grinding of meat during burger processing can decrease the cooking yields due to the breakdown of myofibrils and connective tissue within the meat [49]. The cooking yield of chicken sausages formulated with carrot pomace powder also increased, rising from 98% to 101% and to 104% with the addition of pomace at 6% and 9%, respectively [4]. The high water-holding capacity of carrot pomace sausages not only increased the cooking yield but also improved emulsion stability. Carrot pomace incorporation at 6% and 9% showed a 5% increase in the emulsion stability of sausages [4]. Similarly, adding pea fiber at 1.5% improved the water-holding capacity by up to 15% and cooking yield by up to 4% [27].

#### 3.1.5. Color of Raw Beef Patties

Raw control beef patties showed higher L*, a*, and b* values than beef patties formulated with 1%, 3%, and 4.2% carrot pomace (*p* ≤ 0.05) (Table 3). Although there was a slight decrease in the L* value (Lightness) in the carrot patties as the pomace percentage increased, this change was not significant (*p* > 0.05). Redness (a*) decreased significantly by 40% in the 4.2% pomace patties compared to the control. This reduction in redness could be due to the masking effect or dilution of myoglobin in the pomace patties. There was no significant difference in yellowness (b*) regardless of the pomace level, potentially due to the orange color contribution and additional effects of the carrot pomace.

Functional ingredients can impact the color of meat products due to their pigments and colors [4]. Carrot pomace is rich in carotenoids, which may or may not significantly affect redness and yellowness. Similar results were observed in beef patties made with a 3% pumpkin mix, where the a* values decreased from 14.14 to 8.52 [42]. The level of pomace incorporation and source of meat can have an influence on the degree of color intensity. Unlike beef, chicken sausages with up to 9% dried carrot pomace showed no significant differences in L* and a* values [4].

The 4.2% pomace patties showed the largest ΔE value (5.59), indicating a distinct and noticeable difference compared to the control patty (Table 3). The Δa*(−3.85) and Δb* (−3.18) values were the main contributors to the ΔE in the 4.2% patties. In contrast, the patties with 1% and 3% pomace had ΔE values between 2 and 3.5, indicating no noticeable difference. This could be attributed to the Δa* values (−2.75 and −2.42), which show a shift away from redness to green.

#### 3.1.6. Texture Profile Analysis of Cooked Beef Patties

In texture profile analysis (TPA), hardness-1 and -2 (Kg) indicate the maximum force measured at the first (first bite) and the second (second bite) compressions, respectively. Beef patties with 4.2% carrot pomace showed a significantly lower hardness-1 of 3.47 kg compared to 4.99 ± 1.28 kg for patties with 0% pomace (*p* < 0.05). However, no significant difference in hardness was observed between the control patties and those with less than 4.2% pomace (Table 4). Hardness-2 decreased significantly at 3% and 4.2% pomace additions over the control, with no reduction observed at the 1% addition level. The decrease in hardness could be due to the incorporation of fiber, which improved moisture retention within the beef patty. Similar reductions in hardness were observed when soy hull pectin and insoluble fiber were incorporated into fresh and frozen/thawed beef patties [7].

No significant differences were found in adhesiveness and springiness between the control and carrot pomace treatments (*p* > 0.05) (Table 4). Cohesiveness was reduced from 0.425 to 0.230, but there was no significant difference between the control patties and those with pomace. Patties with 1% pomace were significantly more cohesive than those with 3% and 4.2% pomace (*p* > 0.05). Similar results were seen in beef patties containing 3% carrot mash [50]. Chewiness and gumminess showed a significant difference between the control and patties with 1% or more pomace. Specially, gumminess and chewiness decreased from 2.15 g to 0.80 g and from 165.53 mJ to 70.23 mJ, respectively, as the carrot pomace percentage increased from 0 to 4.2%. Similar results were seen in beef patties containing gari (cassava flour), where gumminess and chewiness decreased from 14.57 to 3.15 and from 65.38 to 14.13, respectively, with a 20% addition [51]. These reductions were primarily due to the decrease in hardness. Chewiness and gumminess are secondary parameters in the texture profile analysis and are influenced by hardness, cohesiveness, and springiness scores. The control patty was significantly (*p* ≤ 0.05) harder, chewier, gummier, and more cohesive compared to the carrot pomace patties. These results are consistent with the expected increase in moisture retention with the addition of carrot pomace.

Different fiber sources have different impacts on the textural properties of meat products. Increased hardness has been seen in dry fermented sausages with the addition of carrot fiber [20], while the incorporation of apple and orange fibers resulted in decreased hardness and increased springiness in low-fat sausages [52]. In the present study, no large significant differences in textural properties were observed between the 1% and 3% pomace patties compared to the control patties. These results indicate that dried carrot pomace can be incorporated into beef patties at levels up to 3% without negatively affecting the quality and textural properties.

### 3.2. Sensory Evaluation of Beef Patties with Carrot Pomace

#### 3.2.1. Consumer Acceptability with and Without a Bun

Table 5 displays the demographic information of 74 of the 86 participants (*n* = 74) who responded to the demographic questionnaire. There was a higher proportion of female participants (*n* = 57; 77%) than male participants (*n* = 15; 20%), with a small number of participants preferring not to mention their gender (*n* = 2; 3%). The 40–49-year age group was the most predominant (*n* = 19; 26%), followed by the 50–59-year age group (*n* = 17; 23%) and the 30–39-year age group (*n* = 14; 19%). These three age groups accounted for 68% (*n* = 50) of all respondents. Respondents who purchased beef once per week and those who purchased it more than once per week accounted for 49% (*n* = 36), while 48% (*n* = 35) purchased beef once every two weeks or once a month among those who responded to the questionnaire. All respondents (*n* = 74; 100%) indicated that they did not have any food allergies.

In this study, no significant differences were observed between the control patties and the 1% or 3% carrot pomace patties for appearance, aroma, taste, chewiness, beef flavor, sweetness, texture, and overall liking (Table 6). However, the appearance score of the 3% carrot pomace patties were slightly higher than that of the control patties (7.27 vs. 6.77), likely due to the increased orange tint provided by the carrot pomace. As the percentage of pomace in the patties increased, the a* value shifted from 9.62 at 0% to 5.76 at 4.2% pomace (Table 3). This was a shift from a red hue to a more orange hue.

No significant differences were found in beef flavor between the control and pomace patties, except for the 4.2% pomace patties, which showed a significant low score. A similar trend was observed with higher levels of non-meat ingredients. For instance, incorporating 15% or 30% soy protein into beef patties reduced the beef flavor and overall acceptability [53]. The 4.2% patties had a significantly lower sweetness score (5.64) than the control patties (6.03). While increased sweetness might have been expected with higher pomace levels, participants might detect off-flavors that masked the sweetness. Off-flavors such as bitterness are a common challenge in plant-based meat products with high levels of replacements [54].

A lower firmness score was found in the pomace patty at 4.2% than those at 1.0 and 3.0%, with an intermediate value observed for the control patty. This aligns with the TPA results, which showed a decrease in hardness values as the level of carrot pomace increased.

As the pomace levels increased in the range of 1–3%, consumer acceptability decreased by more than 3% for most attributes. The top 3 box percentages for all attributes indicated that the changes in hedonic scores can be attributed to a change in the number of participants. Similar findings have been reported in studies where the consumer acceptability of dry fermented sausages decreased as the fiber content increased [20]. Incorporating non-meat ingredients into beef patties at higher-than-ideal levels can negatively affect consumer acceptance [55,56].

#### 3.2.2. Effect of Bun Addition on Sensory Perception of Beef Patties with Carrot Pomace

The 3% pomace beef patties showed the largest relative reduction in taste (−3.12%) and beef flavor (−5.19%) when consumed with a bun (Table 7). Beef flavor decreased in all samples when evaluated in combination with the bun. When evaluated with a bun, the meaty flavor intensity of both soy-based and hemp-based patties was reduced by 14% and 21.1%, respectively [41]. For the sweetness attribute, there was an increase of 2.85% and 3.44% with the 1% and 3% carrot pomace patties, respectively, compared to the control patty with a bun. The inclusion of the bun potentially contributed to an additional perception of sweetness that likely masked the off-flavors of pomace at higher percentages.

Chewiness and juiciness attributes decreased across all beef patty samples when consumed with a bun. Relative juiciness scores decreased by −8.67% in the 3% patties and −7.60% in the 4.2% patties. Juiciness is generally related to a high-quality product and strongly influences the overall liking of meat and meat products such as patties, steaks, and sausages [57,58]. Juiciness perception in meat is influenced by lubrication from fats, oils, water, as well as the stimulation of saliva during chewing. Generally, a decreased juiciness score would be expected with the addition of a dry bun, which absorbs moisture and saliva during consumption. However, the inclusion of a bun did not result in any significant decreases in the overall attributes compared to the patty alone. In fact, likability increased as sensory complexity, texture, and flavor contrast rose with the addition of a bun.

Just-about-right scores reveal that 68% and 75% of panelists found the color of patties, with 1% and 3% pomace, respectively, to be “just about right” when evaluated (Table 8). The desirable color could be attributed to the orange color of the carrot pomace, which provides an increased orange tint. Panelists generally found that samples containing pomace were “not juicy enough”, which was reflected in the overall liking scores of the products. A similar result was observed when the patty was consumed with the bun (Table 9). These juiciness results could have been affected by the reheating process of the samples. Over 60% of participants found that the beef flavor, with and without the bun, was “just about right” in patties containing 1% and 3% carrot pomace. These results indicate that adding carrot pomace up to 3% did not negatively affect the perception of the beef flavor of the samples, nor did it introduce any off flavors.

The penalty analysis indicates how the sensory attributes (color, juiciness, and beef flavor) affected the overall liking of the sample (Table 10). It shows how many points were lost due to a product that was either “too much” or “too little” for a consumer. When it comes to color, it is worth noting that, as the percentage of pomace increased to 4.2%, the participants penalized the product for being “too light”. Beef flavor, with and without a bun, was significantly penalized for being “not strong enough” at higher pomace addition levels of 3% and 4.2% (Table 11). Juiciness, being “not juicy enough”, was also a significant contributor to a decrease in juiciness scores at 3% pomace addition.

## 4. Conclusions

Incorporating carrot pomace into beef patties can enhance their functional properties while addressing food waste concerns. The inclusion of carrot pomace decreases the moisture content, particularly at higher levels (3% and 4.2%). There are no notable changes in the protein, fat, or ash contents across the samples, but the carbohydrate content increases with the addition of carrot pomace. The pomace also causes a color change, especially at the 4.2% level, resulting in a reduction in redness (a*) and yellowness (b*). The cooking yield improves significantly with 3% and 4.2% pomace due to the pomace’s high water-holding capacity (WHC), which enhances water retention during cooking and increases yield. The texture analysis shows that higher pomace levels reduce hardness-1, hardness-2, gumminess, and chewiness, while springiness remains unaffected, and cohesiveness shows only slight changes.

The sensory analysis reveals no significant differences in appearance, aroma, taste, or overall liking between the control patties and those containing 1% or 3% pomace. However, patties with 4.2% pomace receive lower ratings for overall liking, firmness, and beef flavor, likely due to off-flavors introduced at the higher pomace level. When a bun is included, it generally reduces juiciness and beef flavor scores, although overall acceptability remains relatively high for patties with 1% and 3% pomace. Carrot pomace can be successfully incorporated into beef patties at up to a 3% level without significantly compromising sensory attributes or consumer acceptance. However, at 4.2%, the texture and flavor changes become more noticeable, negatively impacting consumer preferences.

## Figures and Tables

**Table 1:** Formulation of beef patties with dried carrot pomace (% *w*/*w*).

Ingredients	Source and Location	0.0%CP ^1^	1.0%CP ^1^	3.0%CP ^1^	4.2%CP ^1^
Beef round, knuckle, lean	Meat Processing Center, California Polytechnic State University	75.60	74.60	72.60	71.40
Beef fat	Meat Processing Center, California Polytechnic State University	15.30	15.30	15.30	15.30
Sea salt, fine	First Street, Smart & Final, Commerce, CA, USA	1.60	1.60	1.60	1.60
Ice	Food science Pilot Plant, California Polytechnic State University	5.75	5.75	5.75	5.75
Black pepper, ground	First Street, Smart & Final, Commerce, CA, USA	0.25	0.25	0.25	0.25
Cultured dextrose	MicroGARD 730, International Flavors & Fragrances, New York, NY, USA	1.00	1.00	1.00	1.00
Onion powder	First Street, Smart & Final, Commerce, CA, USA	0.50	0.50	0.50	0.50
Carrot pomace, freeze-dried	Grimmway Family Farms, Arvin, CA, USA	0.00	1.00	3.00	4.20
Total		100.00	100.00	100.00	100.00

^1^ 0.0%CP = Control beef patty (no pomace); 1.0% CP = Beef patty with 1.0% freeze-dried carrot pomace; 3.0%CP = Beef patty with 3.0% freeze-dried carrot pomace; 4.2%CP = Beef patty with 4.2% freeze-dried carrot pomace.

**Table 2:** Proximate composition, pH, and water-holding capacity of the raw beef patty (on a dry basis) and yield of cooked beef patties at different pomace levels (0%, 1%, 3%, and 4.2%).

	0.0%CP ^1^	1.0%CP ^1^	3.0%CP ^1^	4.2%CP ^1^
Moisture (%)	63.90 ± 0.82 ^a^	63.70 ± 0.18 ^ab^	60.40 ± 0.78 ^c^	61.80 ± 1.03 ^bc^
Protein (%)	18.34 ± 0.55 ^a^	19.18 ± 2.08 ^a^	17.70 ± 1.36 ^a^	17.64 ± 1.06 ^a^
Fat (%)	25.50 ± 2.26 ^a^	24.22 ± 2.29 ^a^	21.94 ± 0.72 ^a^	21.19 ± 1.07 ^a^
Carbohydrates (%)	7.56 ± 1.70 ^b^	7.44 ± 2.20 ^b^	13.13 ± 0.68 ^a^	12.09 ± 1.03 ^ab^
Ash (%)	2.60 ± 0.24 ^a^	2.74 ± 0.30 ^a^	2.94 ± 0.17 ^a^	2.96 ± 0.09 ^a^
Dietary Fiber (%) *	−	0.72	2.15	3.00
pH	5.59 ± 0.02 ^a^	5.48 ± 0.01 ^a^	5.25 ± 0.02 ^a^	5.20 ± 0.05 ^a^
WHC (%)	77.08 ± 4.83 ^b^	78.91 ± 0.82 ^ab^	83.66 ± 0.70 ^ab^	89.16 ± 1.41 ^a^
Cook Yield (%) **	67.47 ± 2.55 ^c^	72.22 ± 1.36 ^bc^	76.85 ± 1.04 ^b^	82.84 ± 2.10 ^a^

^1^ 0.0%CP = Beef patty with 0.0% freeze-dried carrot pomace; 1.0% CP = Beef patty with 1.0% freeze-dried carrot pomace; 3.0%CP = Beef patty with 3.0% freeze-dried carrot pomace; 4.2%CP = Beef patty with 4.2% freeze-dried carrot pomace. ^a–c^ Different letters within the same rows indicate significant differences (*p* ≤ 0.05). * Calculated values not obtained through experimentation [6]. ** Values determined on cooked patties.

**Table 3:** L*a*b* color values of raw beef patties with carrot pomace and color difference of beef patties with carrot pomace compared to a control beef patty.

Sample	L*	∆L*	a*	∆a*	b*	∆b*	∆E
0.0%CP ^1^	53.61 ± 3.39 ^a^	−	9.62 ± 2.82 ^a^	−	11.09 ± 3.48 ^a^	−	−
1.0%CP ^1^	53.75 ± 5.42 ^a^	0.141	6.86 ± 1.64 ^ab^	−2.75	9.46 ± 1.82 ^a^	−1.63	3.21
3.0%CP ^1^	51.60 ± 3.63 ^a^	−2.00	7.20 ± 1.63 ^ab^	−2.42	10.15 ± 2.40 ^a^	−0.94	3.28
4.2%CP ^1^	51.12 ± 8.11 ^a^	−2.49	5.76 ± 2.92 ^b^	−3.85	7.91 ± 4.56 ^a^	−3.18	5.59

^1^ 0.0%CP = Beef patty with 0.0% freeze-dried carrot pomace; 1.0% CP = Beef patty with 1.0% freeze-dried carrot pomace; 3.0%CP = Beef patty with 3.0% freeze-dried carrot Pomace; 4.2%CP = Beef patty with 4.2% freeze-dried carrot pomace. ^a,b^ Different letters within the same physical treatment indicate significant differences (*p* ≤ 0.05).

**Table 4:** Texture profile analysis of cooked beef patties at different pomace levels (0%, 1%, 3%, and 4.2%).

Treatment	Hardness Cycle 1 (kg)	Adhesiveness (mJ)	Hardness Cycle 2 (kg)	Cohesiveness	Springiness (mm)	Gumminess (g)	Chewiness (mJ)
0.0%CP ^1^	4.99 ± 1.28 ^a^	0.79 ± 0.53 ^a^	4.08 ± 1.15 ^a^	0.42 ± 0.11 ^ab^	7.78 ± 1.16 ^a^	2.15 ± 0.72 ^a^	165.53 ± 63.78 ^a^
1.0%CP ^1^	3.69 ± 0.64 ^ab^	0.25 ± 0.20 ^a^	3.21 ± 0.74 ^ab^	0.68 ± 0.56 ^a^	8.44 ± 1.10 ^a^	2.35 ± 1.59 ^a^	206.57 ± 158.34 ^a^
3.0%CP ^1^	3.69 ± 0.64 ^ab^	0.62 ± 0.80 ^a^	2.78 ± 0.47 ^b^	0.24 ± 0.03 ^b^	7.91 ± 0.56 ^a^	0.99 ± 0.16 ^b^	77.33 ± 13.66 ^b^
4.2%CP ^1^	3.47 ± 0.57 ^b^	0.36 ± 0.44 ^a^	2.23 ± 0.56 ^b^	0.23 ± 0.09 ^b^	8.31 ± 1.10 ^a^	0.80 ± 0.31 ^b^	70.23 ± 0.09 ^b^

^1^ 0.0%CP = Beef patty with 0.0% freeze-dried carrot pomace; 1.0% CP = Beef patty with 1.0% freeze-dried carrot pomace; 3.0%CP = Beef patty with 3.0% freeze-dried carrot pomace; 4.2%CP = Beef patty with 4.2% freeze-dried carrot pomace. ^a,b^ Different letters within the same measurement indicate significant differences (*p* ≤ 0.05).

**Table 5:** Demographics of participants (*n* = 74) who responded to the demographic questionnaire.

	Proportion (%)	Base Size *n*
Total	−	74
Gender Group		
Male	20	15
Female	77	57
Prefer not to say	3	2
Age Group		
18–21	9	7
22–44	3	2
25–29	8	6
30–39	19	14
40–49	26	19
50–59	23	17
60 and Over	12	9
Food Allergies		
Yes		
No	100	74
Purchase Frequency		
Never	0	0
Once or twice every three months	4	3
Once a month	26	19
Once every two weeks	22	16
Once per week	34	25
More than once per week	15	11

**Table 6:** Consumer sensory evaluation using a 9-point hedonic scale of carrot pomace beef patties (0%, 1%, 3%, and 4.2%) with and without a bun.

Attributes	0.0%CP ^1^	0.0%CPB ^2^	1.0%CP ^1^	1.0%CPB ^2^	3.0%CP ^1^	3.0%CPB ^2^	4.2%CP ^1^	4.2%CPB ^2^
Appearance Top 3 Box	6.77 ^ab^ 66%	-	7.00 ^ab^ 74%	-	7.27 ^a^ 84%	-	6.53 ^b^ 60%	-
Aroma Top 3 Box	6.77^ab^ 66%	-	6.77 ^ab^ 70%	-	7.07 ^a^ 73%	-	6.42 ^b^ 61%	-
Overall Top 3 Box	7.00^a^ 68%	-	7.11 ^a^ 74%	-	6.93 ^a^ 75%	-	6.18 ^b^ 49%	-
Taste Top 3 Box	7.00 ^a^ 71%	6.92 ^a^ 63%	7.04 ^a^ 71%	7.03 ^a^ 73%	6.95 ^a^ 73%	6.74 ^a^ 67%	6.07 ^b^ 50%	6.17 ^b^ 50%
Firmness Top 3 Box	6.82 ^ab^ 67%	6.89 ^ab^ 68%	7.07 ^a^ 78%	7.05 ^a^ 75%	6.81 ^a^ 64%	6.49 ^bc^ 60%	6.14 ^b^ 51%	6.15 ^c^ 43%
Chewiness Top 3 Box	6.67 ^ab^ 63%	6.81 ^a^ 66%	6.89 ^a^ 70%	6.78 ^a^ 71%	6.68 ^ab^ 64%	6.37 ^ab^ 56%	6.19 ^b^ 50%	5.89 ^a^ 40%
Juiciness Top 3 Box	6.41 ^a^ 58%	6.19 ^a^ 44%	6.38 ^a^ 58%	6.33 ^a^ 58%	5.64 ^b^ 40%	5.19 ^bc^ 34%	5.38 ^b^ 38%	5.00 ^b^ 28%
Beef Flavor Top 3 Box	6.96 ^a^ 67%	6.71 ^a^ 64%	7.00 ^a^ 74%	6.89 ^a^ 74%	6.89 ^a^ 68%	6.55 ^a^ 56%	5.96 ^b^ 44%	5.75 ^b^ 39%
Sweetness Top 3 Box	6.03 ^a^ 37%	6.03 ^ab^ 37%	6.14 ^a^ 38%	6.32 ^a^ 48%	5.90 ^a^ 32%	6.11 ^ab^ 38%	5.64 ^b^ 35%	5.68 ^b^ 31%
Texture Top 3 Box	6.78 ^a^ 62%	6.67 ^a^ 59%	6.81 ^a^ 70%	6.81 ^a^ 67%	6.49 ^ab^ 67%	6.29 ^ab^ 56%	6.00 ^b^ 49%	6.06 ^b^ 50%
Aftertaste Top 3 Box	6.53 ^a^ 55%	6.40 ^a^ 48%	6.59 ^a^ 64%	6.60 ^a^ 56%	6.36 ^a^ 52%	6.21 ^ab^ 51%	5.79 ^b^ 38%	5.75 ^b^ 40%
Overall Liking Top 3 Box	6.90 ^a^ 73%	6.93 ^a^ 67%	7.10 ^a^ 77%	7.00 ^a^ 78%	6.79 ^a^ 68%	6.74 ^a^ 68%	6.22 ^b^ 56%	5.93 ^b^ 47%

^1^ 0.0%CP = Beef patty with 0.0% freeze-dried carrot pomace; 1.0% CP = Beef patty with 1.0% freeze-dried carrot pomace; 3.0%CP = Beef patty with 3.0% freeze-dried carrot pomace; 4.2%CP = Beef patty with 4.2% freeze-dried carrot pomace. ^2^ 0.0%CPB = Beef patty with 0.0% freeze-dried carrot pomace and bun; 1.0% CPB= Beef patty with 1.0% freeze-dried carrot pomace and bun; 3.0%CPB = Beef patty with 3.0% freeze-dried carrot pomace and bun; 4.2%CPB = Beef patty with 4.2% freeze-dried carrot pomace and bun. ^a–c^ Different letters within the same row indicate significant differences (*p* ≤ 0.05).

**Table 7:** Relative intensity change (%) of consumer evaluation using a 9-point hedonic scale of carrot pomace beef patties (0%, 1%, 3%, and 4.2%) combined with a bun compared to patties consumed alone.

Attributes	0.0%CPB ^1^ Relative Change	1.0%CPB ^1^ Relative Change	3.0%CPB ^1^ Relative Change	4.2%CPB ^1^ Relative Change
Taste	−1.16	−0.14	−3.12	1.62
Firmness	1.02	−0.28	−4.93	0.16
Chewiness	2.06	−1.62	−4.87	−5.09
Juiciness	−3.55	−0.79	−8.67	−7.60
Beef Flavor	−3.73	−1.60	−5.19	−3.65
Sweetness	0.00	2.85	3.44	0.70
Texture	−1.65	0.00	−3.18	0.99
Aftertaste	−2.03	0.15	−2.42	−0.70
Overall	0.43	−1.43	−0.74	−4.89

^1^ 0.0%CPB= Beef patty with 0.0% freeze-dried carrot pomace and bun; 1.0% CPB= Beef patty with 1.0% freeze-dried carrot pomace and bun; 3.0%CPB = Beef patty with 3.0% freeze-dried carrot pomace and bun; 4.2%CPB = Beef patty with 4.2% freeze-dried carrot pomace and bun.

**Table 8:** Just-about-right scores for carrot pomace beef patties (0%, 1%, 3%, and 4.2%) with and without a bun.

Color	0.0%CP ^1^	1.0%CP ^1^	3.0%CP ^1^	4.2%CP ^1^
Too Dark	4%	7%	19%	14%
Just About Right	58%	68% *	75% *	56%
Too Light	38%	25%	5%	31%
**Juiciness**	**0.0%CP ^1^**	**1.0%CP ^1^**	**3.0%CP ^1^**	**4.2%CP ^1^**
Too Juicy	5%	4%	0%	3%
Just About Right	51%	52%	34%	31%
Not Juicy enough	44%	44%	66%	67%
**Beef Flavor**	**0.0%CP ^1^**	**1.0%CP ^1^**	**3.0%CP ^1^**	**4.2%CP ^1^**
Too Strong	18%	11%	11%	6%
Just About Right	66% *	68% *	64% *	47%
Not Strong enough	16%	21%	25%	47%

^1^ 0.0%CP = Beef patty with 0.0% freeze-dried carrot pomace; 1.0% CP = Beef patty with 1.0% freeze-dried carrot pomace; 3.0%CP = Beef patty with 3.0% freeze-dried carrot pomace; 4.2%CP = Beef patty with 4.2% freeze-dried carrot Pomace. * Indicates a significant just-about-right percentage.

**Table 9:** Just-about-right scores for carrot pomace beef patties (0%, 1%, 3%, and 4.2%) with and without a bun.

With Bun Juiciness	0.0%CPB ^1^	1.0%CPB ^1^	3.0%CPB ^1^	4.2%CPB ^1^
Too Juicy	1%	3%	0%	1%
Just About Right	45%	42%	25%	25%
Not Juicy Enough	53%	55%	75%	74%
**With Bun Beef Flavor**	**0.0%CPB ^1^**	**1.0%CPB ^1^**	**3.0%CPB ^1^**	**4.2%CPB ^1^**
Too Strong	12%	8%	5%	4%
Just About Right	68% *	70% *	60% *	47%
Not Strong enough	19%	22%	34%	49%

^1^ 0.0%CPB = Beef patty with 0.0% freeze-dried carrot Pomace and bun; 1.0% CPB= Beef patty with 1.0% freeze-dried carrot pomace and bun; 3.0%CPB = Beef patty with 3.0% freeze-dried carrot pomace and bun; 4.2%CPB = Beef patty with 4.2% freeze-dried carrot pomace and bun. * Indicates a significant just-about-right percentage.

**Table 10:** Penalty analysis * for beef patties with carrot pomace (0%, 1%, 3%, and 4.2%) without a bun.

Attribute	Anchors:	0.0%CP ^1^	1.0%CP ^1^	3.0%CP ^1^	4.2%CP ^1^
Color	Too light				0.502 **
Beef Flavor	Not strong enough			0.539 **	1.014 **

^1^ 0.0%CP = Beef patty with 0.0% freeze-dried carrot pomace; 1.0% CP = Beef patty with 1.0% freeze-dried carrot pomace; 3.0%CP = Beef patty with 3.0% freeze-dried carrot pomace; 4.2%CP = Beef patty with 4.2% freeze-dried carrot pomace. * Insignificant-weighted penalty scores are not listed. ** Indicates a significant-weighed penalty score.

**Table 11:** Penalty analysis * for beef patties with carrot pomace (0%, 1%, 3%, and 4.2%) with a bun.

Attribute	Anchors:	0.0%CPB ^1^	1.0%CPB ^1^	3.0%CPB ^1^	4.2%CPB ^1^
w/bun Juiciness	Not juicy enough			0.513 **	
w/bun Beef Flavor	Not strong enough		1.004 **	0.57 **	

^1^ 0.0%CPB = Beef patty with 0.0% freeze-dried carrot pomace and bun; 1.0% CPB= Beef patty with 1.0% freeze-dried carrot pomace and bun; 3.0%CPB = Beef patty with 3.0% freeze-dried carrot Pomace and bun; 4.2%CPB = Beef patty with 4.2% freeze-dried carrot pomace and bun. * Insignificant-weighted penalty scores are not listed ** Indicates a significant just-about-right percentage.

## Data Availability

The data presented in this study are available upon request from the corresponding author. The data are not publicly available due to privacy restrictions.

## Supplementary Materials

The following supporting information can be downloaded at: https://www.youtube.com/watch?v=X7mJP_MCR8U. Video S1: Beef patty reheating instructions.

## Appendix A

### Beef Patty Preparation for Sensory Evaluation

Things you will need:
  - Four-quart pot with lid
  - 3.5 quarts water
  - Scissors
  - Plates

1. If you do not intend to test your hamburger patty when you arrive home, please put the sample in the refrigerator.
2. Fill a four-quart pot with 3.5 quarts of water, place it on stove and turn it on high.
3. Now remove one patty from refrigerator or cold storage bag, the samples will be in vacuum packed bags.
4. Once the water has reached a boil turn the water down below the boil.
5. Now select hamburger patty ###. Place the vacuum-sealed bag containing the hamburger patty in hot water. Please do not mix up samples.
6. The bags should remain in the water for 5 min at a simmer to ensure that they are heated up to an internal temperature of 165 °F
7. After 3 min have expired, remove the vacuum-sealed pack from the water. Please be cautious when removing the bags, as they will be hot. Use an oven mitt if possible.
8. Allow it to cool for 3 min then cut the bag with scissors.
9. Now you may remove the hamburger patty from the bag and place it onto a plate.
10. Now please reference the green sheet on how to conduct the sensory panel.
11. Each patty will be tested individually with a 5-min break between completion of the questionnaire.
